# Reconstructing high fidelity digital rock images using deep convolutional neural networks

**DOI:** 10.1038/s41598-022-08170-8

**Published:** 2022-03-11

**Authors:** Majid Bizhani, Omid Haeri Ardakani, Edward Little

**Affiliations:** 1 Natural Resources Canada, Geological Survey of Canada, 3303 33 Street NW, Calgary, AB T2L 2A7 Canada; 2 Department of Geoscience, University of Calgary, 2500 University Drive NW, Calgary, AB T2N 1N4 Canada

**Keywords:** Imaging techniques, Scanning electron microscopy, Fossil fuels, Geology, Geophysics, Imaging and sensing

## Abstract

Imaging methods have broad applications in geosciences. Scanning electron microscopy (SEM) and micro-CT scanning have been applied for studying various geological problems. Despite significant advances in imaging capabilities, and image processing algorithms, acquiring high-quality data from images is still challenging and time-consuming. Obtaining a 3D representative volume for a tight rock sample takes days to weeks. Image artifacts such as noise further complicate the use of imaging methods for the determination of rock properties. In this study, we present applications of several convolutional neural networks (CNN) for rapid image denoising, deblurring and super-resolving digital rock images. Such an approach enables rapid imaging of larger samples, which in turn improves the statistical relevance of the subsequent analysis. We demonstrate the application of several CNNs for image restoration applicable to scientific imaging. The results show that images can be denoised without a priori knowledge of the noise with great confidence. Furthermore, we show how attaching several CNNs in an end-to-end fashion can improve the final quality of reconstruction. Our experiments with SEM and CT scan images of several rock types show image denoising, deblurring and super-resolution can be performed simultaneously.

## Introduction

Convolutional neural networks (CNN) have brought about substantial improvements in a variety of image transformation problems such as single image super-resolution^1–3^, denoising^4–6^, deblurring^7,8^, and segmentation^9,10^. Deep learning models can learn complex, non-linear priors over the image domain that makes them particularly powerful for restoration tasks. Of particular interest to the scientific imaging, are the problems of image denoising, deblurring, and super-resolution. The former two have been studied for decades, and sophisticated non-linear algorithms exist that can restore the underlying texture with great confidence^11,12^. Super-resolution is a relatively new concept that has largely been enabled through the application of CNNs.

CNNs have found applications in many engineering problems, such as microcosmic events identification^13^, remote sensing^14^, self-driving cars^15^, healthcare system^16,17^, signal processing and image defect detection^18,19^, and many more examples. In this work, we aim to explore the application of such networks in pre-processing and automating the workflow of using digitial rock images.

In the study of geologic formations, imaging methods such as scanning electron microscopy (SEM), micro-CT scanning, and optical and fluorescence microscopy are commonly applied for different purposes^20^. Collectively, the use of digital images for geoscientific studies and in particular fluid flow-related properties are dubbed as digital rock physics. Current technologies allow imaging specimens at unprecedented resolutions^21^. 4D imaging (i.e.  imaging through time) has also been successfully demonstrated for dynamic problems^22,23^. Rapid CT scan imaging is partially enabled by multi-GPU aided reconstruction of X-ray attenuation maps^24^.

Digital rock physics has two bottlenecks. The first is the trade-off between resolution and sample size. High-resolution imaging is limited to a small sample size, which affects the statistical relevance of the subsequent analysis. Increasing sample size can result in the loss of smaller sub-resolution features, which can once again change the underlying properties. The second problem is imaging speed, which becomes problematic when studying dynamic problems such as fluid flow. Acquiring large SEM images takes days to weeks as each image must be sampled many times for noise reduction. Techniques such as focused ion beam (FIB) combined with SEM can take a long time to produce small high-resolution 3D images^12^. Micro-CT scanner also suffers from the same problem, where if the goal is to image moving objects, the resolution will be affected adversely due to the sampling rate.

A solution to the previous problems is to obtain low-resolution large images at higher speed and restore the underlying high-resolution noise-free image through image processing. Concurrently, scientific image restoration demands high-fidelity accurate reconstruction. Unlike video or image enhancement tasks for recreational purposes, where perceptual properties are of importance, in scientific imaging, accuracy of the underlying distribution is critical. It is at this forefront that our research tries to contribute to the current body of literature. We study three fundamental image restoration tasks including denoising, deblurring and super-resolution of rock sample SEM and CT scan images using deep CNNs.

The SEM or CT images of rock specimen often have varying levels of noise. Due to the nature of these methods, varying levels of energy are used for imaging, hence, causing quality degradation as well as noise level. High electron power (or X-ray intensity) can provide a clearer image, but at the cost of damaging the sample^25^. In some cases, high-speed imaging is required. In such cases, due to lower power and dwell time the resultant image is of low-resolution and corrupted with noise; similar situations are encountered in satellite image processing.

The blurring of images occurs mostly due to non-ideal point spread function (PSF), or when imaging moving targets. This happens mostly in optical microscopy of living cells. Deconvolution (e.g.  Richardson–Lucy) is the most commonly used image processing method in optical imaging systems to remove the blur. While this method has been successful in deblurring, it suffers from several disadvantages, such as slow processing time due to multiple iterations required to deblur and sub-optimal PSF^7^.

Finally, image resolution is often the limiting factor in studying micro-structures. CT scanner can provide 3D realization of internal structures of opaque materials. However, the resolution of the current industrial CT scanner is insufficient for resolving most sub-micron features. The latter is important in the study of tight rock samples such as carbonates or shales.

Boosting image resolution can be performed using deep CNNs. Single image super-resolution has been widely studied over the past 5 years^26–29^. Super-reslolution CNN (SRCNN) was the first shallow CNN that demonstrated this concept^30^. Many studies have since developed with the particular aim of boosting resolution. Most image transformation tasks, and in particular image super-resolution is an ill-posed problem where multiple realizations of a given low-resolution image could be the answer. Therefore, scientific image super-resolution must be rigorously validated to ensure the underlying statistics conform to the ground truth. Note that super-resolved image refers to high-resolution image produced from a low-resolution image, i.e.  using CNNs, while high-resolution is used for ground truth images that were acquired from an imaging instrument.

In this study, we aim to restore high-fidelity images of various rock samples from a given low-resolution and corrupt SEM or CT scan image. The main objective is to explore the accuracy of reconstruction through state-of-the-art CNNs. Eventually, we want to perform image denoising, deblurring and super-resolution in one single model. We experiment with various types of CNNs. The methods and results are equally applicable to other types of scientific images such as medical images, and satellite imagery. The scope of our work is limited to 2D images.

## Data

Training deep neural networks requires large datasets. Furthermore, supervised learning requires pairs of input and ground truth for the training. Obtaining training pairs in scientific imaging is challenging, as often ground truth does not exist. Researchers often resort to synthetic data for training deep neural networks. We also employ this technique because it provides greater control over the training data. We note that capturing complex non-linear degradation that occurs in the real-world imaging cannot be accurately replicated synthetically. However, we try to make the process of image degradation as close to the real-world as possible.

In this work, we use $\approx$27,000 image patches of size $256\times 256$ pixels (e.g.  ground truth images) for training and testing each model. The data contains images of organic-rich shale of the Vaca Muerta formation, Bentheimer sandstone, and the bioclastic carbonate Estaillades limestone, all equally distributed in numbers. The data are obtained through the DigitialRocks portal^31–33^.

The shale images were obtained using the FIB-SEM method with a resolution of 2.5 nm/pixel^34^. The Bentheimer sandstone and Estaillades carbonate are CT scan images. The sandstone was imaged at 2.25 $\upmu$m/pixel^35^, and the carbonate at 3.9 $\upmu$m/pixel^36^. Each sample rock have $\approx$ 30,000 images. We randomly sample around 9000 images for each rock type and mix them to create the training dataset.

The mix of data from three rock types, and methods of imaging represent increasing structural complexity in data. The sandstone images are clear, with high contrast features, whereas the shale sample has a texture with high-frequency features. The choice of these three formation types are partly driven by our research interest about porous rocks that can be used for CO_2_ storage, and or have potential fluid production. Other rock types such as coal can be added to the dataset in a similar fashion. Please see the “Methods” section for sample training images of the three rock types.

To obtain training image pairs required for supervised learning, we first downsample the images using the local mean method, then blur the images using a Gaussian blur kernel with random variance in the range of [0, 2], and finally add a random amount of Gaussian, Poison, and speckle noise to the blurred images. We note that in real-world cases, a low-resolution image will be corrupted by noise and other environmental factors that cannot be reproduced by downsampling a high-resolution image. Our procedure adds noise and blur to the downsampled image to produce as close to a real low-resolution image as possible. Details of the image preparation procedures are discussed in the “Methods” section.

Three metrics are used for assessing the quality of image reconstruction, namely peak-signal-to-noise-ratio (PSNR), structural similarity index measure (SSIM), and multi-scale SSIM. Further detail on the reconstruction metrics are presented in the “Methods” section.

## Results

As per the objective of the study, our goal is to recover a high-fidelity image from a corrupted low-resolution input. Since the images are downsampled, blurred and corrupted by various noises, we approach the problem sequentially at first. In the first part of the results section, we discuss the performance of CNNs on each individual task. In the last section, we explore the application of end-to-end approaches in reconstructing the final target image directly from the noisy input. Finally, we test the developed networks on an unseen dataset to test their generalization capability.

### Image denoising

In denoising, the aim is to remove noise and restore the true image. However, since noise, edge, and texture are high-frequency components, it is difficult to distinguish them in the process of denoising; the denoised images could inevitably lose some details^11^. Traditional denoising methods such as non-local means filter, Gaussian filtering or wavelet thresholding often smooth the image, and require a priori knowledge of the noise type and an estimate of the noise amount.

Digital images are corrupted with various types of noises. In scientific imaging, noise can come from a variety of sources. Poisson noise originates from the varying number of electrons that hit the specimen at each measurement spot. Gaussian noise is the result of microscope electronics^25^. Speckle noise rarely a problem for SEM or CT scan imaging, however, we add this type of noise to our images to further complicating the denoising task.

Denoising through filters assumes a particular type of noise or requires iterative procedures to estimate the denoiser’s parameters. Gaussian noise is the most prevalent in which ${\hat{y}} = y + \mathcal {N}(0, \sigma )$, where *y* is the true image, and ${\hat{y}}$ is the image containing noise. If $\sigma$ of the distribution is known, the true image can be recovered. In reality, the distribution is not known, and that is if we assume a Gaussian white noise. SEM images are often corrupted by Poisson noise as well as Gaussian and rarely speckle noise (the latter is mostly observed in satellite images).

Denoising using CNNs has been the target of many studies^37^. While most of these models perform well on their dataset, they often have known noise amount and type in their images (e.g. Gaussian noise of known distribution). Our dataset is corrupted with a random amount of Gaussian, Poisson and speckle noise. The deep iterative down-up CNN, or DIDN for short, devised by Yu et al.^4^ is the main framework adopted here for denoising. Details of the network architecture are discussed in the “Methods” section as well as the supplementary materials. The DIDN network denoises images through a series of convolutional blocks. The network has the capability to adapt to different noise types and amounts, hence, presenting an optimal case for our study. We train the network for minimization of the L1 (i.e. mean absolute error (MAE)) loss. The model is trained for as long as the validation loss of the network stops improving for 5 consecutive epochs. Further detail on the training aspects of the network is presented in the “Methods” section.

**Figure 1:**
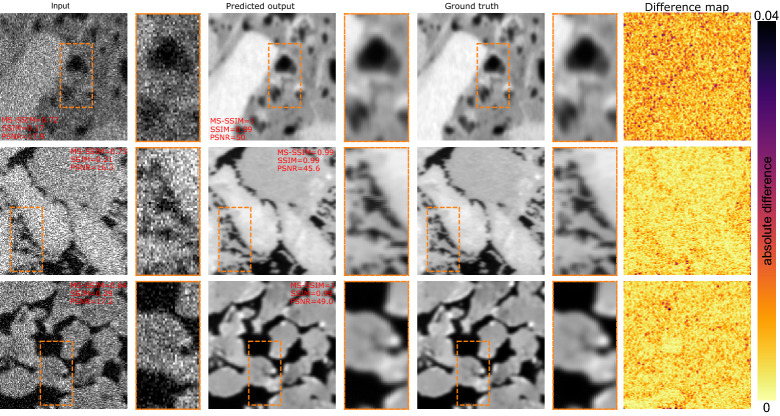
Sample images denoised using DIDN network. The orange rectangular box shows the highlighted area with a zoom factor of 2$\times$. Top row is a sample shale image, middle row is a limestone, and the bottom row a sandstone image. The row order of the rock-type is retained in Figs. 3, 4, 6, 7 and 8.

Figure 1 shows a sample output of the denoiser network. The input images have varying levels of noise. The CNN network successfully reconstructs the target clean images. For the shale image, we see the network increases the PSNR from 17.9 to 50 dB, similarly both MS-SSIM and SSIM increase dramatically. Note that the images in Fig. 1 are from the test data. The last column of Fig. 1 is the absolute difference between the prediction and the ground truth. The difference maps indicate the exceptional performance of the DIDN for denoising.

**Figure 2:**
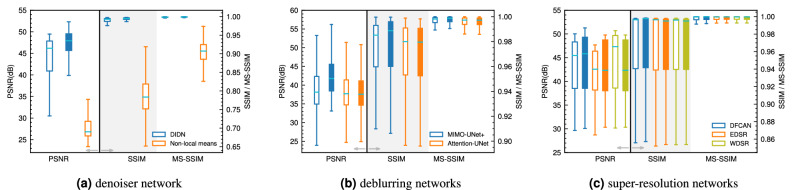
Reconstruction metrics for (**a**) the denoiser network, (**b**) deblurring networks, and (**c**) super-resolution networks.

The performance of the denoiser network in terms of PSNR, SSIM and MS-SSIM on both the training and test data are reported in Fig. 2a. For comparison purposes, we also report denoising performance of the non-local means filter (window size of 7, and search window size of 11 were used). For the CNN network, the training data’s PSNR is mostly larger than 41 dB (based on the 2^nd^ quartile of the boxplot). The results on the test data show improvement over the training data, which may indicate the test data are easier to denoise. We note that the selection of train-test data is random, and the images are shuffled before doing so. Additionally, the varying noise level in each image can contribute to the performance differences between the test and train data. The non-local means filter’s performance is far below the CNN model. This performance degradation is partly due to the unknown noise level and type and in part due to the algorithm used by the non-local means denoising.

The network’s performance in terms of SSIM and MS-SSIM is exceptional. For SSIM, a median value of $\approx$ 0.993 is achieved, while MS-SSIM is $\approx$ 0.999. Both of these metrics indicate a highly efficient reconstruction in terms of structural similarity.

The analysis of the metrics of image reconstruction indicates the network learns to effectively denoise the images. Given the randomness of the noise (type and amount), it is clear that this deep CNN is highly effective in removing noisy signals from images. Furthermore, by training with respect to the L1 loss, we avoid the smoothing of the finer features of the images that are often associated with traditional filtering methods or the L2 loss.

### Image deblurring

Having denoised the images, the next task is to recover a deblurred image from the blurry denoised images. Conventional deblurring methods (i.e. deconvolution) require iterative procedure and an estimate of the point spread function. Similar to denoising, such information is not readily available.

For the deblurring task, we test two CNNs, both having a UNet architecture. The first network uses channel attention and residual blocks and is a combination of models proposed by^38,39^. This network is denoted as Attention-UNet. The second network is a variant of the UNet model proposed by Cho et al^8^. This network is called MIMO-UNet+. We train both networks for minimization of the L1 loss. More detail on the architecture of the MIMO-UNet+ and training is reported in the supplementary materials. We note that the choice of UNet architecture opens up many possibilities for designing the deblurring network. CNN models such as DeepLabv3^40,41^ have exceptional performance in segmentation tasks. However, we only test two networks here that have been specially designed for deblurring tasks.

Figure 2b compares the performance of each network on both the train and test data in terms of the three reconstruction metrics we applied in this study. We observe that MIMO-UNet+ consistently performs better on all three metrics compared to the UNet with channel attention. The MIMO-UNet+ model produces a PSNR roughly higher than 35dB (2^nd^ quartile) on both train and test data. Structural similarity is higher than 0.96, while MS-SSIM is nearly 1 for this network. Further analysis in this paper uses the MIMO-UNet+ for deblurring.

**Figure 3:**
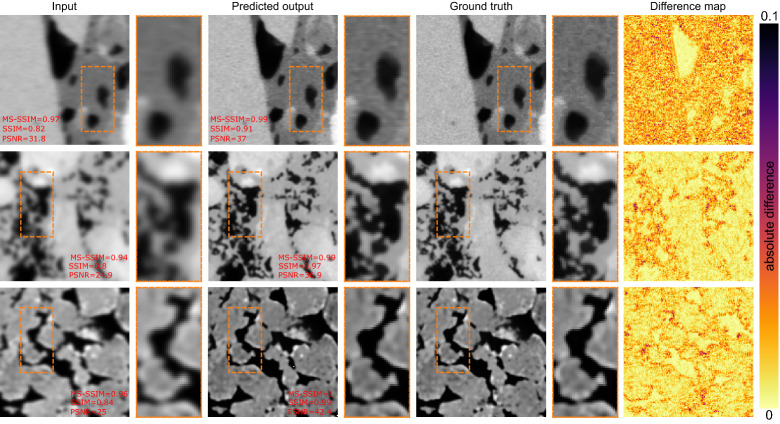
Prediction of the debluring task using the MIMO-UNet+ network.

Figure 3 demonstrates three sample images de-blurred using the MIMO-UNet+ network. The good performance of the network on this task is reflected in these images as well in the associated metrics for each reconstruction. The sandstone images (bottom row) are easier to deblur, while due to high-frequency features in the texture of the shale samples (top row), the performance is slightly impaired. The difference maps show slightly worse performance compared to the denoiser network. Additionally, for the shale sample, the texture is more difficult to reconstruct. The carbonate image (the middle row) is blurred with higher variance, and the results indicate the good performance of the network in recovering a deblurred image.

Overall, comparing to deconvolution methods, CNNs have superior performance both in terms of quality of reconstruction and ease of use. In deconvolution methods, a point spread function and multiple iterations are required for deblurring. Using CNNs, there will be no need for user intervention, as the results indicate the performance is satisfactory to good.

### Image super resolution

Image super-resolution aims to reconstruct high-resolution realization of a given low-resolution image. In the context of scientific images, it is important we recover true features, especially edges.

For super-resolving images, various network architectures have been developed (e.g.  SRCNN^30^, EDSR^42^, WDSR^43^, SRGAN^44^, ESRGAN^45^, DFCAN^46^, RCAN^47^, among many others). A recent paper by Qiao et al.^46^ discussed the use of Fourier transformation for accurate reconstruction of high-frequency features of microscopic images. We experimented with all the mentioned networks and found that EDSR (enhanced deep super-resolution network), WDSR (wide activation deep super-resolution network), and DFCAN (deep Fourier channel attention network) consistently performed better than the other models in terms of reconstruction metrics. We therefore present results of the super-resolution task using the ESDR, WDSR and DFCAN models only. It is noteworthy to mention that GAN (generative adversarial neural networks) based methods perform worse than their regular variant for our purposes. The reason for this could be the training losses that are used in GANs, which push the solution towards a more visually identical image rather than constructing high-frequency features accurately. Details on the architecture and training procedures are reported in the supplementary materials.

EDSR and WDSR are trained with respect to L1 loss, while DFCAN is trained using a weighted L2 and SSIM loss. All three networks increase resolution by a factor of 2.

Figure 2c reports the performance of the three selected super-resolving networks. We note that both EDSR and WDSR networks have $\approx$ 11–13 times the number of trainable parameters compared to DFCAN. The PSNR of the reconstructed high-resolution images is broadly similar, with the WDSR marginally performing better on the training data.

In terms of SSIM, DFCAN shows slight improvement over WDSR, possibly owing to the use of Fourier transformation that results in better reconstruction of high-frequency features. Nonetheless, all three networks perform exceptionally well on this task. Finally, the MS-SSIM for all three networks approaches 1, indicating a near-perfect score for this metric.

Comparing the performance of each network and their computational costs, the DFCAN model outperforms the other two. This network has more than 10 times fewer trainable parameters, and it performs equally or even marginally better on the reconstruction metrics. Therefore, for further experimenting, we use the DFCAN for the super-resolution task.

**Figure 4:**
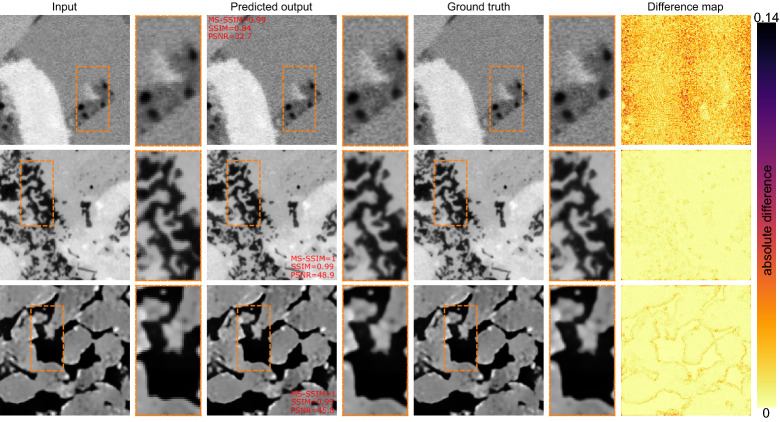
Sample images super-resolved using the DFCAN network (magnification factor of 2).

Figure 4 shows some sample low-resolution images super-resolved using the DFCAN model. Note for the shale samples (first row), the network restores the boundaries accurately. The difference map is the absolute difference between the super-resolved and ground truth images (pixel-wise difference). For the sandstone and carbonate, the difference map indicates highly efficient performance by the network. For shale, there appear to be scattered differences with no particular focus on specific regions of the image. The randomness in difference map of the shale texture is likely due to the inherent noise-type high-frequency features in the ground truth SEM images.

## Experiments

As per the objective of the study, our goal is to reconstruct a high-resolution output given the low-resolution, blurry and noisy input. In the previous sections, we did this by passing the images through separate networks, where each network performed one task. In this section, we examine the performance of the networks where no intermediate outputs are produced.

In order to reconstruct a high-resolution image, we can progressively pass the output of each network to the next network to finally obtain the high-resolution image. In this scheme, we first denoise the image, then the result is deblurred, and finally super-resolved. The networks are not re-trained. In experiment 1 we examine the quality of the final output using this approach.

Another solution for end-to-end processing is to combine all the networks in one “super” model. In this approach, all three tasks are performed simultaneously. Furthermore, the new network is trained to improve the performance. Experiment 2 uses this approach.

### Experiment 1: Stacked model

In experiment 1 we stack all three networks to perform denoising, deblurring and super-resolution on a given low-resolution image. Each network simply takes the output of the previous model as input. The models’ parameters are from the previously trained tasks.

**Figure 5:**
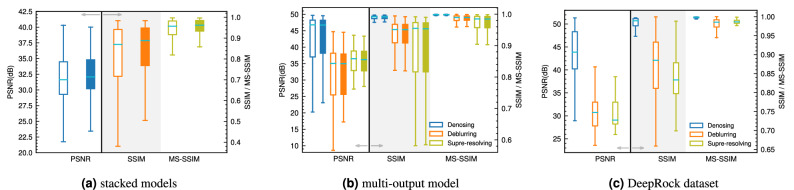
Reconstruction metrics for (**a**) stacked models, (**b**) multi-output model, and (**c**) the DeepRock dataset.

Figure 5a shows the statistics for the three reconstruction metrics for the final super-resolved image given the noisy image input to the stacked network. In terms of PSNR, the stacked model’s performance drops sharply compared to DFCAN model trained separately (see Fig. 2c). PSNR drops by more than 10 dB. Both SSIM and MS-SSIM also drop in this fashion, indicating the inadequacy of this approach.

**Figure 6:**
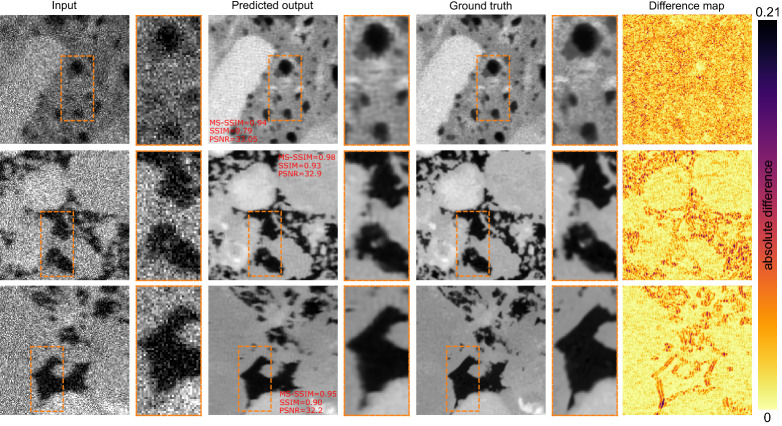
Prediction of super-resolution images using stacked model approach.

Figure 6 shows sample prediction results using the stacked model on the test data. Note how the predicted output is not as sharp as the ground truth (reflected through the difference maps as well). Generally, the drop in PSNR and SSIM both point to the inadequacy of this approach. In the stacked fashion, where each network was trained separately, the model fails to take into account the imperfections in the prediction of the previous networks. Therefore, the final results lack the necessary quality both perceptually and structurally.

### Experiment 2: Multi-output model

In the second experiment, we create a “super” model by combing all three networks (DIDN, MIMO-UNet+, and DFCAN) into one. The resulting network takes one input (noisy, blurry image), and has three outputs, namely denoised, deblurred, and super-resolved image; see the supplementary data for more detail. Each sub-network is instantiated from previously trained weights to accelerate training time. The main difference of experiment 2 with 1 is that in experiment 2 we re-train the networks to optimize for the desired output.

The end-to-end network is compiled using three loss functions, L1 loss for the first two outputs, and a combination of L2 and SSIM loss for the DFCAN network. We train the network for an additional $7\times 10^{4}$ iterations over the mini-batches.

The metrics of reconstruction for the end-to-end model is reported in Fig. 5b. We report the metrics for all three outputs. In terms of denoising, the network’s performance is as good as DIDN trained separately. PSNR values higher than 38 dB (for the test data) and SSIM and MS-SSIM are nearly 1 for the denoising task. The performance for deblurring drops slightly, nonetheless, both SSIM and MS-SSIM indicate a highly efficient performance on this task.

The new model produces super-resolved images with a PSNR in the range of 26–38 dB. The median for both SSIM and MS-SSIM is well over 0.9. Overall, the new network outperforms the previous stacked models’ performance from experiment 1. However, performance slightly drops compared to each individual network. The culprit is the training data. Previously the networks had to learn the image transformation directly from the simulated data. Here, the last network only sees the output of the previous two models as input, and as per the performance of those networks, the performance of the last network is affected.

**Figure 7:**
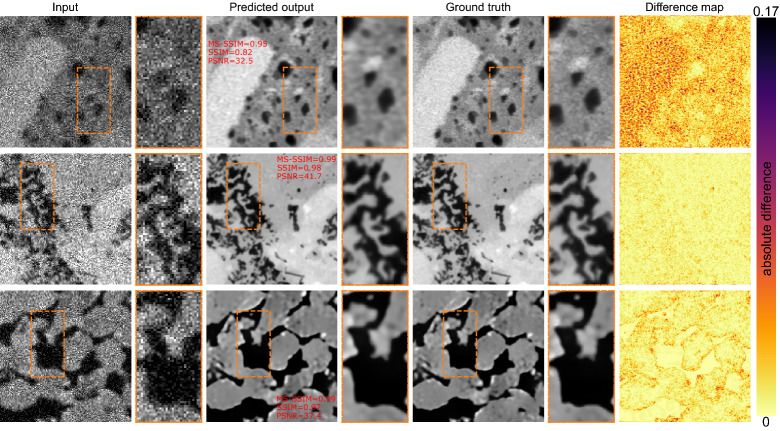
Results of super-resolving low-resolution noisy and blurry images in an end-to-end fashion using multi-output model approach.

Figure 7 shows some sample super-resolved images using the multi-output network. Both the reconstruction metrics and the difference maps indicate the multi-output network performs better than the stacked networks.

The new end-to-end network is more representative of the final performance of deep learning models for the task of super-resolution. In training individual networks, the training data are synthetic, hence, we see very high reconstruction metrics. However, for the end-to-end model, the network has to learn the errors of the previous networks as well, hence, harder to train. In reality, the situation is similar to experiment 2. Raw data are often noisy and low-resolution. A network that performs denoising, and super-resolution can also introduce complex non-linear degradation at small scales. Therefore, whether the introduced artifacts can be tolerated depends on the particular applications in question.

### Experiment 3: Testing the CNN models on unseen data

In the final experiment, we apply the trained CNNs on a different dataset that contains various types of carbonate and sandstone images. The new dataset is from the DeepRock super-resolution project^48^. We used 800 images of sandstone and carbonate (400 each) to create a new test dataset. The low- and high-resolution pairs are from the original authors, we only use smaller patches than the original images. The data were previously used by Wang et al^49^. in their study of image super-resolution. Noisy and blurry images are prepared using the same procedure described previously. We note that the new data contain images with different textures and contrast, hence, present a more challenging task for the pre-trained networks.

The metrics of reconstruction on the DeepRock data are shown in Fig. 5c. In terms of denoising, the DIDN networks perform exceptionally well on this new dataset. All three reconstruction metrics we used indicate a highly efficient denoising performance. The deblurring network (MIMO-UNet+) performs worse than the original training data. PSNR ranges from 23 to 40 dB, with 75% of the images having PSNR over 28 dB. SSIM is also widely spread over a range of 0.65–1. MS-SSIM is higher than 0.95, which is believed to be more representative of the performance of the deblurring network.

For the super-resolution network (DFCAN), the PSNR values are in the range of 27–40 dB. Comparing to Wang et al^49^. results, the DFCAN improves the PSNR range. This is an important point because the DFCAN network was not trained on this dataset, however, it performs better than the original models tested by Wang et al^49^. We further show SSIM, MS-SSIM, which indicate the satisfactory performance of the DFCAN. In particular, MS-SSIM is in the range of 0.97–1, which is a sign of the superior performance of this network.

**Figure 8:**
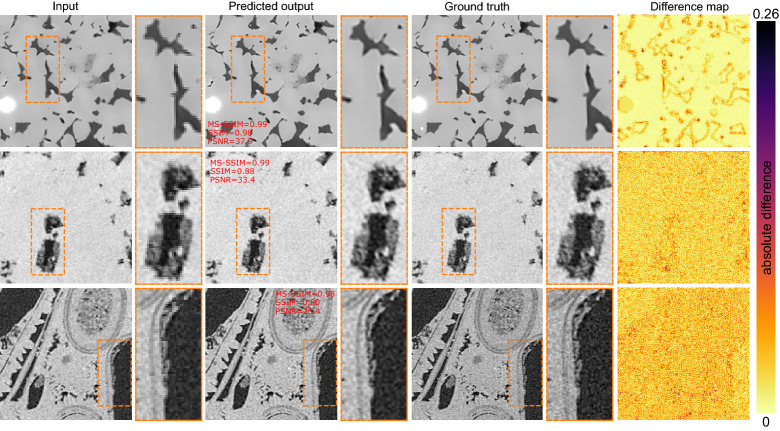
Results of super-resolution network (DFCAN) on the DeepRock dataset.

Figure 8 shows three sample images from the DeepRock dataset super-resolved using DFCAN. The images show different textures and contrast compared to the data used during training the DFCAN. We observe the images are successfully super-resolved using the network. Perceptually, there is little difference between the prediction and the ground truth.

## Discussion

We explored the application of several CNNs for three image reconstruction tasks of denoising, deblurring and single image super-resolution. The dataset was a combination of SEM and CT scan images of shale, limestone and sandstone. The training data was prepared using randomly added noise and blur amounts to downsampled images. Our target was to investigate the performance of deep CNNs in recovering accurate structures from corrupted low-resolution images. We further experimented with another dataset of similar images to explore the performance degradation of trained models.

In terms of image denoising, our analysis shows CNNs with residual learning have exceptional capabilities in recovering a denoised image. The training data had varying levels of Gaussian, Poisson and speckle noise. The denoiser CNN showed very high reconstruction metrics both on our training data as well as the DeepRock dataset. Based on our findings, we conclude a carefully designed CNN can be highly efficient on image denoising for scientific image reconstruction. The benefit of such an approach is in the applicability of CNNs for blind denoising. Trained CNNs do not require assumptions about the noise, hence, are straightforward in their use.

Deblurring is a more challenging task than denoising. A blurred image has high PSNR (compared to the ground truth). The use of MSE loss can results in a blurry realization of a low-resolution image, yielding a false indication that a given CNN is reconstructing the image with accuracy. In our work, we blurred the images using a Gaussian kernel with random multivariate variance. Using two CNNs with UNet architecture and residual learning, we showed deblurring can be done effectively using CNNs. The final results showed high structural similarities with the ground truth images.

Single image super-resolution was performed using three state-of-art CNNs. A channel attention network with the Furrier transformation layer was shown to be superior both in terms of performance and computational cost to other networks. Furthermore, this network was trained using a weighted L2 and SSIM loss to produce high-resolution images with high SSIMs. Overall, despite being an ill-posed problem, the reconstruction metrics showed satisfactory performance of the super-resolution network for 2$\times$ magnification improvements.

In the last section of the paper, we explored the application of end-to-end networks for producing high resolution images directly from low-resolution, blurry and noisy images. In the first attempt, we sequentially fed the output of each individual network to the next in order to produce clean high-resolution images. Reconstruction metrics in this model showed sub-optimal performance. The sequential approach of processing images results in complex image degradation (caused by each model) that eventually manifests itself in the form of low performance of reconstruction of the final output.

To address the reconstruction issues of the stacked models, we experimented with combining all three image transformation tasks into a single “super” model. The resultant network has one input and three outputs. The network was trained with respect to three losses. The performance metrics of the new end-to-end model indicate better performance on the final super-resolution task. The MS-SSIM in particular demonstrated greatly improved outputs as compared to the stacked model approach.

Given the results, we conclude that it is best to perform image reconstruction in an end-to-end fashion where all the networks are trained simultaneously. Such a scheme results in the networks better adapting to complex image degradation caused by other models and yields better metrics. This is equally applicable to other image transformation tasks such as denoising and deblurring. We suggest a sub-network be dedicated to each task.

Finally, performance is highly dependent on the training data. If one wishes to use deep learning for image enhancement, it is imperative that similar images have been used for model training and validation. Texture has less of an impact than luminosity and contrast for the purpose of the model’s generalization capability. We tested several CNN architectures, and reported the best preforming models for each task. For application of these models to other datasets, we strongly recommend using similar images (e.g.  images with similar texture to our training dataset). If the models are to be used for completely different rock types (e.g.  granite), we recommend training the models using these data through transfer learning. Image denoising and deblurring can be applied to other datasets, however, image super-resolution may require retraining to generalize to other rock types.

On the applicability of deep CNNs for scientific image reconstruction, it is dependent on the specific objectives of the study. If the goal is to rapidly image a sample, carefully designed deep learning models can significantly improve the output, and improve the subsequent analysis. Conversely, if the goal is to study the smallest microstructures (such as protein structures or nano-pores in shales), then one must proceed with caution. Furthermore, different reconstruction tasks have different performances. Image denoising is easier than deblurring and super-resolution.

The limitations of our work mostly lies with the data. Most deep learning models are trained on synthetic data, where the mapping of training to output data is known. In reality, quality degradation is complex. In our work, we added noise and blur to a downsampled images to re-create a more realistic task of reconstructing the high-resolution image. However, obtaining real-world training data for each network can substantially improve the confidence in their capability. Additionally, we only considered 2D images. Applicability of our approach for 3D images needs to be tested.

## Conclusions

In this study, we used several deep CNNs to perform a number of image reconstruction tasks commonly used in microscopic and medical image processing. Our analysis indicates image denoising, deblurring and super-resolution can be performed with great confidence using state-of-art CNNs. Image denoising is the easiest of the three transformation tasks for CNNs. A deep CNN with residual learning architecture is best suited for this task.

In the image super-resolution, leveraging frequency domain rather than spatial domain yields better performance for scientific image processing.

Finally, our results indicate if the goal is to recover a high-resolution image, it is best to perform denoising, deblurring and super-resolution all in one model trained simultaneously. Performing the tasks separately causes a rippling effect throughout the reconstruction pipeline that eventually manifests itself in reduced confidence of reconstruction.

## Methods

### Data

For preparing the training data no pre-processing is applied on the raw images, i.e.  the downloaded images are treated as ground truth for reconstruction. To obtain low- and high-resolution image pairs, the images are downsampled using the local mean method. The local mean method replaces each pixel with the mean value of pixels in the desired window size. This method is more representative of real low-resolution realization than bi-cubic interpolation. The images are downsampled by a factor of 2.

To simulate non-linearity in the image artifacts, we apply a Gaussian blur kernel to the downsampled images to further degrade the image quality. Each image is blurred using a different kernel, with the sigma of the multivariate distribution randomly chosen in the range of $\sigma \in [0, 2]$. The $\sigma$ of the kernel is randomly chosen in each direction. The resultant images are randomly blurred, which we then use for training the deblurring network.

Finally, noisy-clean image pairs were obtained by adding random Gaussian, Poisson, and speckle noise to the blurred images. Similar to the blurring task, to add more randomness to the dataset, we use a random variance for the noise kernel in the range of $\sigma \in [0{-}30]$. The mean value of the Gaussian distribution of the noise is zero. In each image, all three types of noises are present. The noisy-blurred image pairs are used to train a denoiser network.

**Figure 9:**
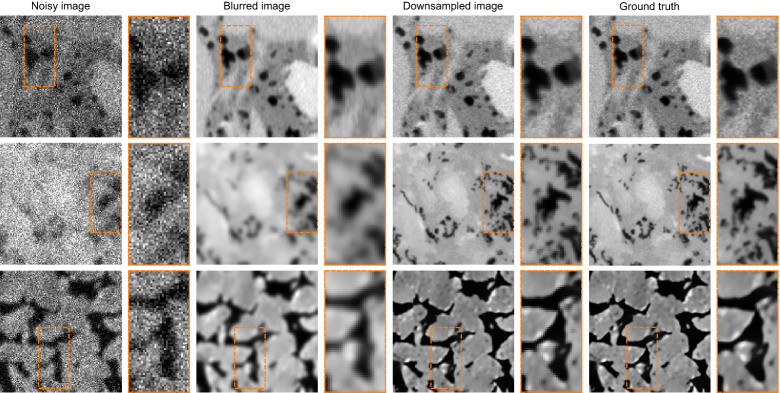
Sample training image pairs. The orange rectangular box shows the highlighted area with a zoom factor of 2$\times$. Top row is a sample shale image, middle row is a limestone, and the bottom row a sandstone image.

Figure 9 shows three sample training images, one for each rock type. The top row is a typical shale image. Note the texture in the high-resolution image contains high-frequency features, reminiscent of noise. Additionally, organic matter has an intensity close to those of pores in these images, which can make pore detection difficult. The goal is to reconstruct the high-resolution image given the noisy and corrupt input. Note that each blurry and noisy image contains a random amount of blurring or noise. We note that only downsampling the images wont be representative of a real world low-resolution realization of the high-resolution image. In reality, the noisy, blurred low-resolution image is the more likely image obtained at low-resolution. Having said that, our dataset is synthetic, hence, may not represent all the complex artifacts experienced in real world cases. Our method in which noise and blur of random amount and types are added to downsampled images is an attempt of recreating a realistic low-resolution image.

Of the $\approx$ 27,000 images used in this study, we reserved 10% of them for testing purposes. The remaining 90%, comprised 10% used for validation data during training and 80% used for supervised training. The data are shuffled before splitting to ensure equal distribution from all three image types in the training and test dataset.

### Reconstruction quality metrics

To quantify the quality of reconstruction, we use three separate metrics: (1) peak signal-to-noise ratio (PSNR); (2) structural similarity index measure (SSIM), and (3) multi-scale structural similarity index measure (MS-SSIM).

Peak signal-to-noise ratio is often used as the main metric in image reconstruction tasks, and the higher its value, the better the reconstruction quality. The PSNR calculation only takes into account the mean squared error (MSE) difference between the images. Since PSNR relies on the MSE, its value can be high despite large differences in the two images. In particular, it is known that the use of L2 (i.e. MSE) loss for CNNs results in overly smooth/blurry images with high PSNR. PSNR is a measure of the maximum signal strength (max possible pixel intensity) to the corrupting noise. Equation (1) is used for the calculation of PSNR.

1 $$\begin{aligned} \text {PSNR} = 10 \log _{10}\left( \frac{\max (I)^{2}}{\text {MSE}} \right) = 20\log _{10}(\max (I)) - 10\log _{10}(\text {MSE}) = -10\log _{10}(\text {MSE}) \end{aligned}$$

In Eq. (1), $\max (I)$ is the maximum pixel value, which is equal to 1 for normalized images. MSE is the mean squared error difference between the two images. The final form of the equation shows PSNR in our application only depends upon the final MSE.

Structural similarity index measure and MS-SSIM are perceptual metrics and are more stringent measures of the reconstruction quality. Factors such as luminosity and contrast are considered in the calculation of these metrics. Comparing to PSNR, these metrics are more representative in the domain of scientific images. SSIM, on the other hand, is also affected by small illumination differences in the images and can lead to some misleading results.

The structural similarity index (SSIM) extracts 3 key features for computing the metric. These features are luminance, contrast, and structure^50^. For images, x and y with size $N\times N$, SSIM is computed according to Eq. (2).

2 $$\begin{aligned} \text {SSIM} = \left[ [l(x,y)]^{\alpha } [c(x,y)]^{\beta } [s(x,y)]^{\gamma } \right] \end{aligned}$$

In Eq. (2), *l*(*x*, *y*), *c*(*x*, *y*) and *s*(*x*, *y*) are measurements of luminance, contrast and structure comparison functions defined according to Eqs. (3)–(5).

3 $$\begin{aligned}&l(x,y ) =\frac{2\mu _{x}\mu _{y} + c_{1}}{\mu _{x}^{2}+\mu _{y}^{2}+c_{2}} \end{aligned}$$

4 $$\begin{aligned}&c(x,y ) =\frac{2\sigma _{xy}+c_{2}}{\sigma _{x}^{2}+\sigma _{y}^{2}+c_{2}} \end{aligned}$$

5 $$\begin{aligned}&s(x,y ) =\frac{\sigma _{xy}+c_{3}}{\sigma _{x}\sigma _{y}+c_{3}} \end{aligned}$$

In Eqs. (3)–(5), $\mu$ and $\sigma$ are defined using the following equations.

6 $$\begin{aligned}&\mu _{x} = \frac{1}{N}\sum _{i=1}^{N}x_{i} \end{aligned}$$

7 $$\begin{aligned}&\sigma _{x} =\left( \frac{1}{N-1}\sum _{i=1}^{N} (x_{i} - \mu _{x})^{2} \right) ^{\frac{1}{2}} \end{aligned}$$

8 $$\begin{aligned}&\sigma _{xy} =\frac{1}{N-1}\sum _{i=1}^{N} (x_{i} - \mu _{x})(y_{i} - \mu _{y}) \end{aligned}$$

$c_{3} = c_{2}/2$, $c_{1} = (K_{1}L)^{2}$, $c_{2} = (K_{2}L)^{2}$, L = 1, and $K_{1}$ and $K_{2}$ are 0.01 and 0.03 respectively.

In Eq. (2), $\alpha =\beta =\gamma = 1$. We note that the above formulation is applied to smaller patches of the given images, and the overall average of the SSIM is computed for the final SSIM score.

Multi-scale structural similarity index measure computes the SSIM at several scales, as such it is tuned to provide a better estimate of the structural similarity at multiple scales^51^. For calculation of MS-SSIM we use a filter size of 7, and a Gaussian blur kernel with $\sigma =1.5$. The formal definition of MS-SSIM is presented in Eq. (9).

9 $$\begin{aligned} \text {MS-SSIM} = \left[ [l(x,y)]^{\alpha _{M}} \prod _{j=1}^{M} [c(x,y)]^{\beta _{j}} [s(x,y)]^{\gamma _{j}} \right] \end{aligned}$$

In the MS-SSIM definition, $\alpha _{M} = \beta _{M} = \gamma _{M}$ for $\sum _{j=1}^{M} \gamma _{j} = 1$. In this study we use the default values provided by the original authors (i.e.  M=5, and $\alpha =\beta =\gamma$ = [0.0448, 0.2856, 0.3001, 0.2363, 0.1333]).

**Figure 10:**
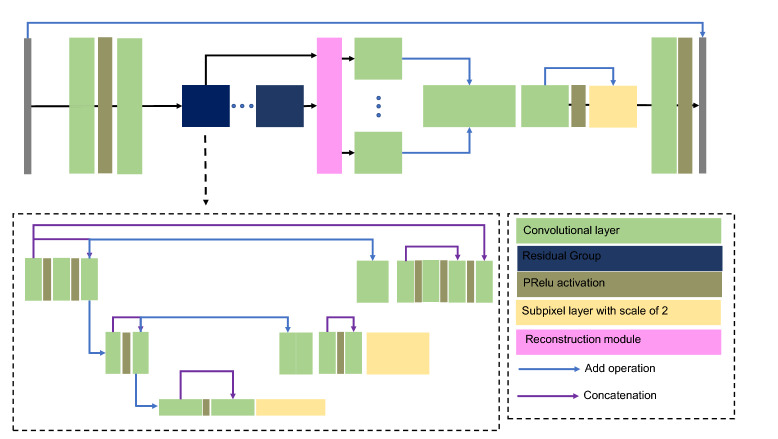
Architecture of the DIDN network used for image denoising (after^4^).

### Training details

All the CNNs in this study were implemented using using Keras and TensorFlow 2 as its backend. We use a multi-step learning rate schedule with an initial learning rate of $10^{-4}$, and reducing by a factor of 0.5 down to a minimum learning rate of $1.25\times 10^{-5}$ for all the networks. Adam optimizer is used for updating networks’ parameters. Each network is trained for as long as the pre-defined loss function does not reduce for 5 consecutive epochs. All the networks in this study were trained on a GPU instance using the AWS cloud computing service. Below we present the training details for the denoiser network, similar information for other CNNs are reported in the supplementary materials.

#### Denoising network

The denoiser network is adopted from Yu et al^4^. The network uses a deep iterative down-up CNN (DIDN) architecture. The DIDN has residual blocks along with skip connections. The architecture of the model is shown in Fig. 10. We use 48 feature maps for the initial convolutional block. Two residual groups are used. The final model has $\approx$ 22 million trainable parameters. Other model parameters such as kernel size are similar to the original model proposed by^4^. A mini-batch size of 16 is used for training the denoiser CNN. The denoiser network is trained with respect to the L1 loss, or the mean absolute error (MAE).

**Figure 11:**
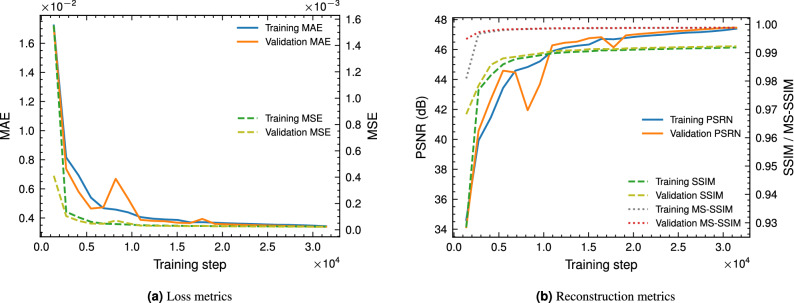
Loss and reconstruction metrics of the DIDN network during training and validation.

Figure 11 reports the loss and reconstruction metrics of the DIDN network during training. The plots for the validation data exhibits the model is not overfitting the training data. Furthermore, the L1 (MAE) loss plateaus after $\approx 2.5\times 10^{4}$ mini-batch iterations. We also report the MSE loss during training for comparison purposes.

## Supplementary information

Supplementary Information.

## Data Availability

The dataset generated and analyzed during the current study is available online through Digital Rocks Portal.
