# Supplementary material for: Reconstructing high fidelity digital rock images using deep convolutional neural networks
Source: Sci Rep. 2022 Mar 11;12:4264. doi: 10.1038/s41598-022-08170-8 (PMC8917167; doi:10.1038/s41598-022-08170-8)
Supplement: Supplementary file 1 — Supplementary Information. [file 41598_2022_8170_MOESM1_ESM.pdf]

Reconstructing high fidelity digital rock images using deep convolutional neural networks - supplementary data

Majid Bizhani[1,*], Omid Haeri Ardakani[1,2], and Edward Little[1]

[1]Natural Resources Canada, Geological Survey of Canada, 3303 33 Street NW, Calgary, Alberta, T2L 2A7, Canada
[2]Department of Geoscience, University of Calgary, 2500 University Drive NW, Calgary, Alberta, T2N 1N4, Canada
[*]majid.bizhani@NRCan-RNCan.gc.ca

ABSTRACT

Imaging methods have broad applications in geosciences. Scanning Electron Microscopy (SEM) and micro-CT scanning have been applied for studying various geological problems. Despite significant advances in imaging capabilities, and image processing algorithms, acquiring high-quality data from images is still challenging and time-consuming. Obtaining a 3D representative volume for a tight rock sample takes days to weeks. Image artifacts such as noise further complicate the use of imaging methods for the determination of rock properties with confidence. In this study, we present applications of several convolutional neural networks (CNN) for rapid image denoising, deblurring and super-resolving digital rock images. Such an approach enables rapid imaging of larger samples, which in turn improves the statistical relevance of the subsequent analysis. We demonstrate the application of several CNNs for image restoration applicable to scientific imaging. The results show that images can be denoised without a priori knowledge of the noise with great confidence. Furthermore, we show how attaching several CNNs in an end-to-end fashion can improve the final quality of reconstruction. Our experiments with SEM and CT scan images of several rock types show image denoising, deblurring and super-resolution can be performed simultaneously.

Models

All the CNNs in this study were implemented using using Keras and TensorFlow 2 as its backend. We use a multi-step learning rate schedule with an initial learning rate of 10^{-4}, and reducing by a factor of 0.5 down to a minimum learning rate of 1.25×10^{-5} for all the networks. Each network is trained for as long as the pre-defined loss value does not reduce for 5 consecutive epochs. All the networks in this study were trained on a GPU instance using the AWS cloud computing service. Below we present the training details for the deblurring and super-resolution networks, similar information about the denoiser network is presented in the methods section of the main manuscript.

Deblurring network

For image deblurring, we use two networks, as outlined in the main manuscript. However, since the performance of the UNet network with channel attention mechanism is not as good as the other UNet network, we only discuss details of the latter network here. The deblurring task is done using a variant of the MIMO-UNet+ model proposed by Cho et al.[1]. The architecture of the model is schematically shown in Fig. 1. The model's parameters are similar to the original authors' proposal. We use an initial number of filters equal to 32, and 20 residual blocks in each residual group. The final model ≈21 million trainable parameters.

The deblurring CNN is trained with respect to L1 loss. Figure 2 reports the training loss and metrics for the MIMO-UNet+ network. We also report the MSE or the L2 loss for comparison purposes. Low values of L2 loss results in high PSNR.

Deep Fourier channel attention network (DFCAN)

The super-resolution network adopted in this study is the deep Fourier channel attention network (DFCAN) from the work of Qiao et al.[2]. The architecture of the network is schematically shown in Fig 3. We use the same parameter settings as those originally proposed by the authors. The final network has ≈1.2 million parameters.

As per the suggestion of the authors of the DFCAN model[2], we use a weighted L2-SSIM loss for training the network. Equation 1 is the final loss function used for the super-resolution network. The parameter λ is set to 0.1 here.

$$\text{loss} = \text{MSE}(y, \hat{y}) + \lambda \left[1 - \text{SSIM}(y, \hat{y})\right] \tag{1}$$

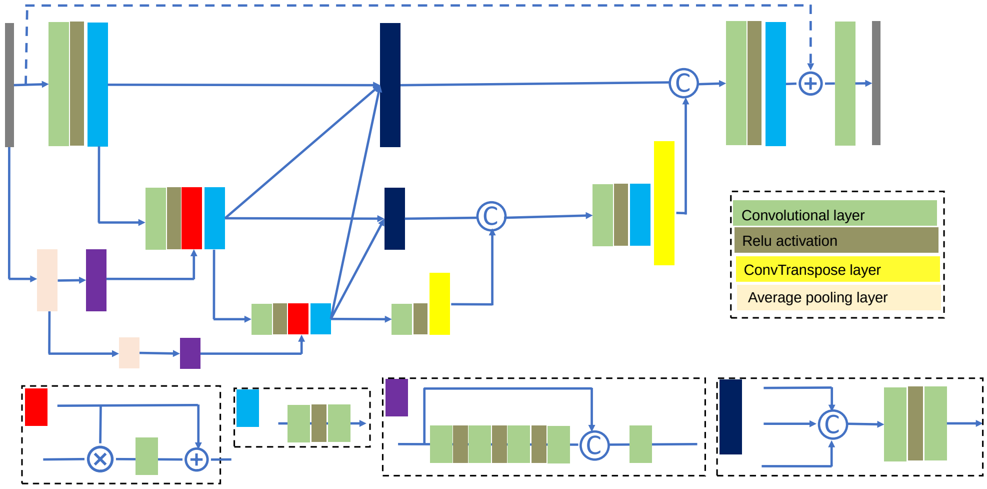

Figure 1. Architecture of deblurring network (after[1])

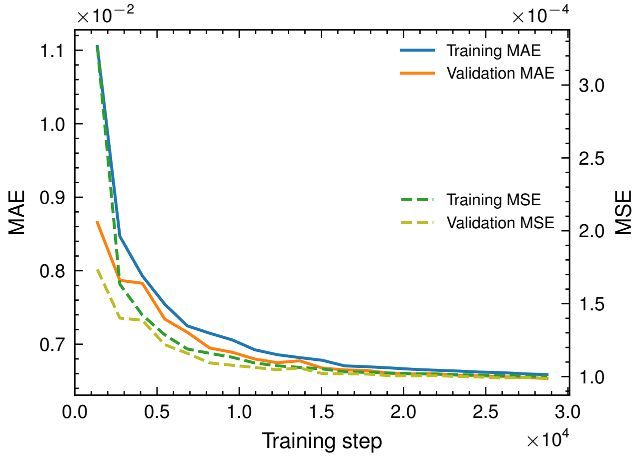

(a) Loss metrics

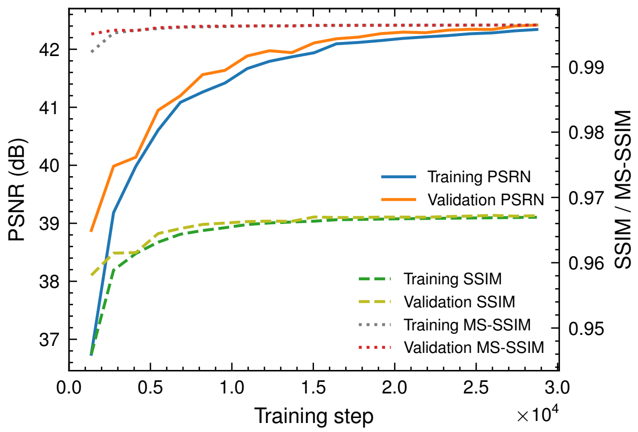

(b) Reconstruction metrics

Figure 2. Loss and reconstruction metrics of the MIMO-UNet+ network during training and validation.

In Eq. 1 y and $\hat{y}$ are the ground truth image and the super-resolved image, respectively. Training loss and metrics for DFCAN model is shown in Fig. 4. The loss for the training set converges after $\approx 2 \times 10^4$ steps. However, PSNR curves show an asymptotic behaviour, which suggests slight improvements of the results is possible by training for more steps.

Multi-output model

The architecture of the multi-output model is shown in Fig. 5. Note the input is the low-resolution noisy and blurry image. The model is compiled with three losses, L1 loss for the first two outputs (i.e. the denoiser and deblurring networks), and the weighted L2-SSIM loss for the super-resolution CNN. We further note that the multi-output model is trained on 4 GPUs in a distributed manner.

Loss and reconstruction metrics during training the multi-output model are reported in Fig. 6. We report the metrics for each sub-networks separately, as well as the total loss of the super network. As the curves show, the loss values for the DFCAN and MIMO-UNet+ may be reduced further by longer training times. The reconstruction metrics, particularly MS-SSIM, plateau long before the losses.

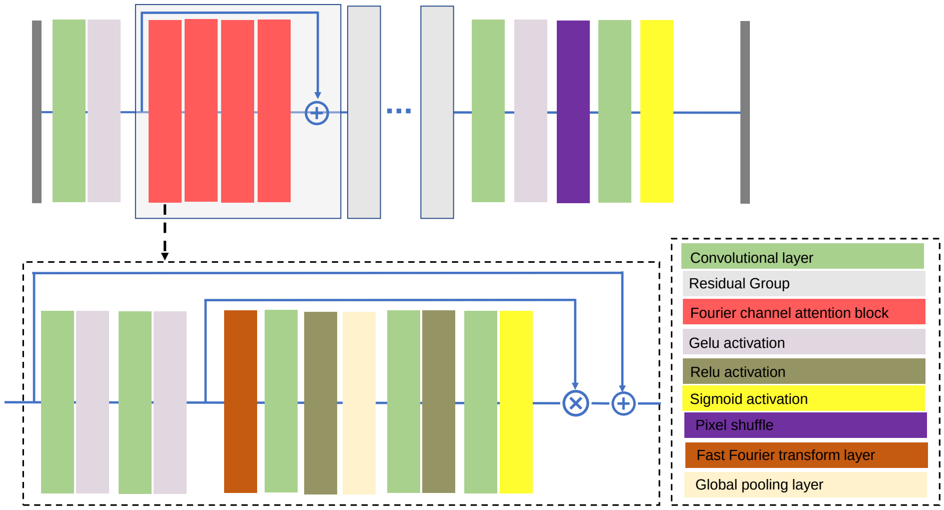

Figure 3. Architecture of the DFCAN network used for super-resolution (after[2])

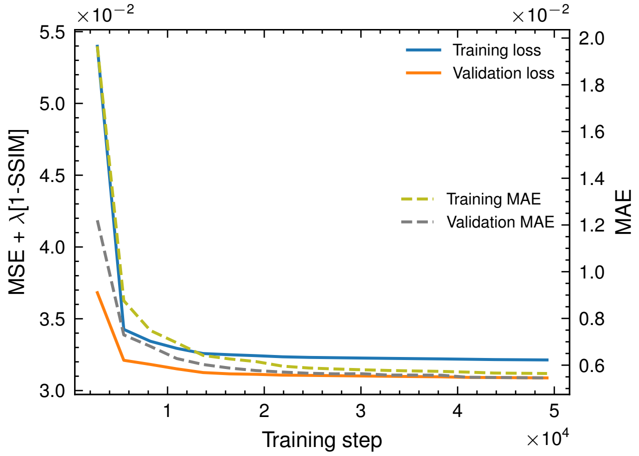

(a) Loss metrics

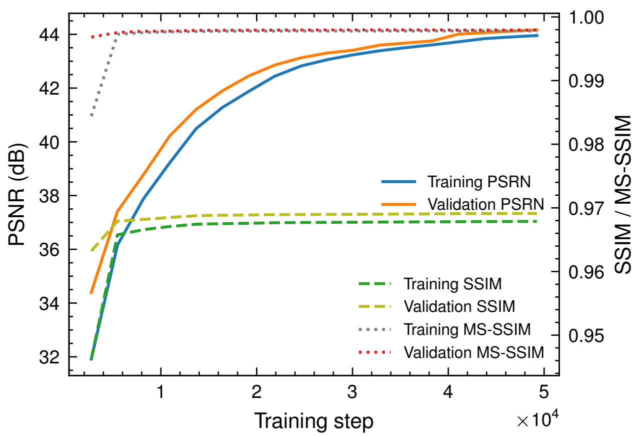

(b) Reconstruction metrics

Figure 4. Loss and reconstruction metrics of the DFCAN network during training and validation.

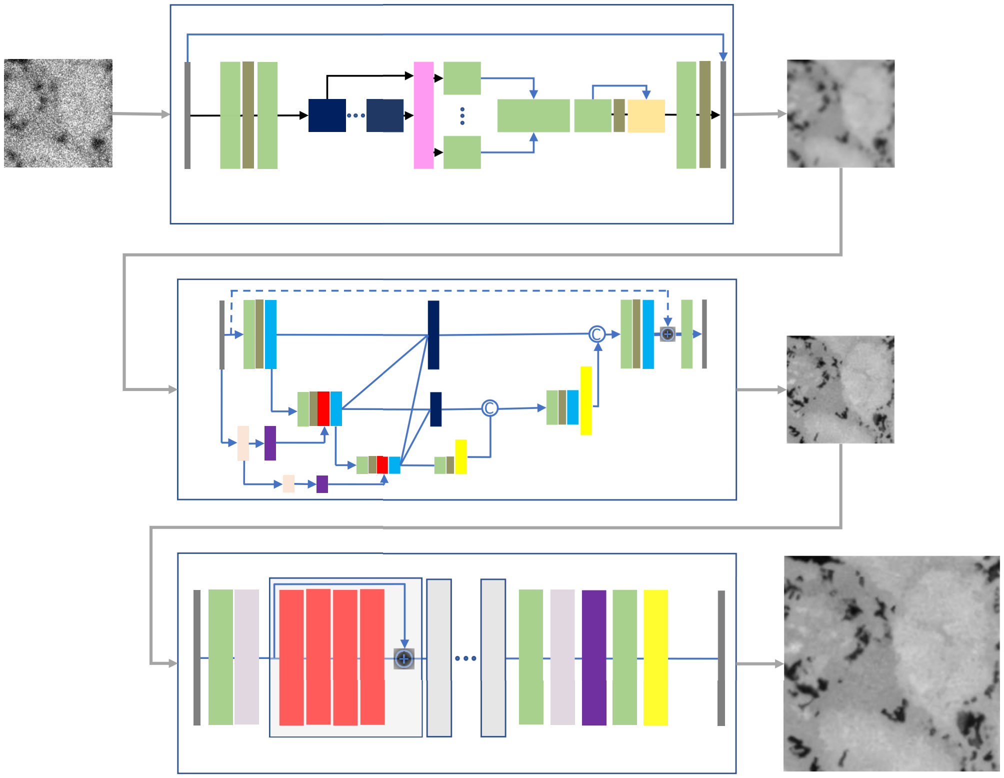

Figure 5. Architecture of the multi-output network with three sub-modules for image denoising, deblurring, and super-resolution

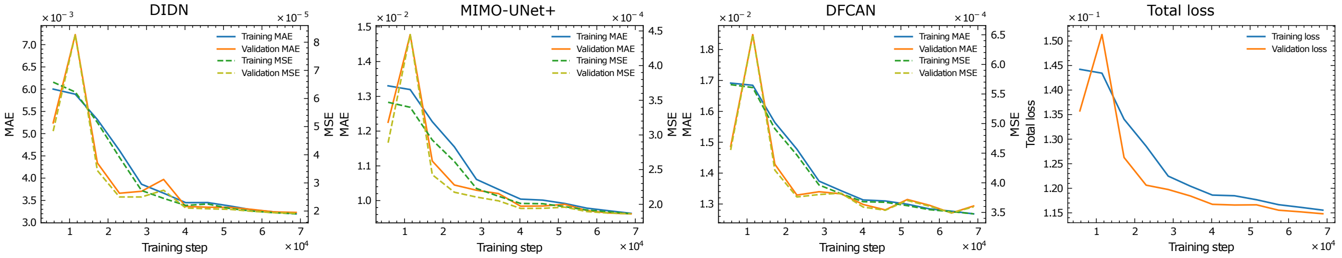

(a) Loss metrics

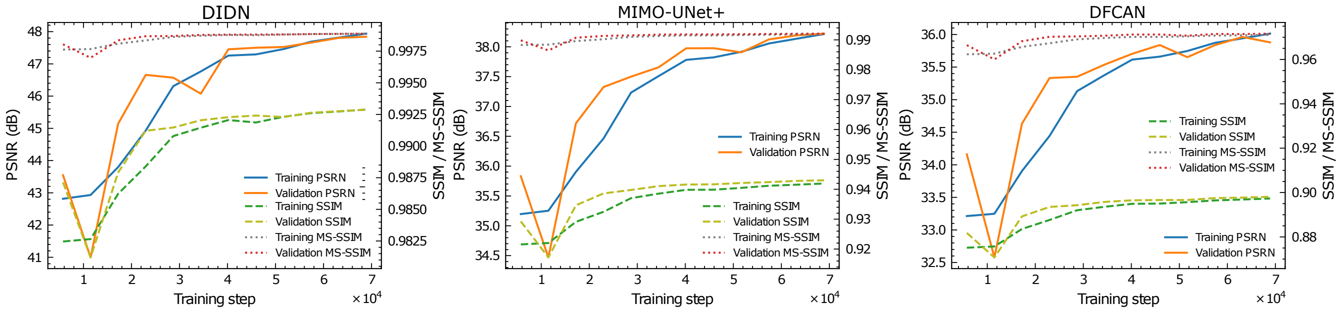

(b) Reconstruction metrics

Figure 6. Loss and reconstruction metrics of the multi-output network during training and validation. Note total loss is the sum of losses for the three networks.

References

1. Cho, S. J., Ji, S. W., Hong, J. P., Jung, S. W. & Ko, S. J. Rethinking Coarse-to-Fine Approach in Single Image Deblurring (2021). https://arxiv.org/abs/2108.05054.

2. Qiao, C. *et al.* Evaluation and development of deep neural networks for image super-resolution in optical microscopy. *Nat. Methods* **18**, 194–202, DOI: https://doi.org/10.1038/s41592-020-01048-5 (2021).
